# Pharmacological targeting of one‑carbon metabolism as a novel therapeutic strategy for glioblastoma

**DOI:** 10.1186/s12967-023-04185-5

**Published:** 2023-06-29

**Authors:** Yanfei Sun, Guangjing Mu, Zhiwei Xue, Xingang Li, Xiaoying Lin, Mingzhi Han

**Affiliations:** 1 Department of Neurosurgery, Qilu Hospital, Cheeloo College of Medicine and Institute of Brain and Brain-Inspired Science, Shandong University; Jinan Microecological Biomedicine Shandong Laboratory and Shandong Key Laboratory of Brain Function Remodeling, Jinan, China; 2 The Second Hospital, Cheeloo College of Medicine, Shandong University, Jinan, China; 3 Medical Integration and Practice Center, Cheeloo College of Medicine, Shandong University, Jinan, China

## Dear Editor,

Glioblastoma multiforme (GBM) is the most malignant central nervous system brain tumor. Despite advances in surgical and medical neuro-oncology, the median survival rate is only 15 months after the first diagnosis and with standard surgery followed by concurrent radiotherapy and chemotherapy [1]. To develop more effective therapeutics, it is critical to understand the molecular pathways that contribute to GBM formation and progression.

Methylenetetrahydrofolate dehydrogenase 2 (MTHFD2) is one of the major enzymes involved in mitochondrial folate one-carbon metabolism. Despite its widely recognized bifunctional dehydrogenase and cyclohydrolase activities, MTHFD2 has been demonstrated to be essential for cancer proliferation and may play a significant role in tumor formation and progression. In GBM, the process of serine-dependent one-carbon metabolism is abolished with knockdown of MTHFD2, causing tumor cell death, especially under glutamine starvation [2]. Recently, since potent and specific inhibitors of MTHFD2 have been developed [3, 4], it is important to validate the translational significance of these findings in a pharmacological context.

To determine the antitumor effects of MTHFD2-based druggable dependencies, we used the following two novel MTHFD2 inhibitors: (i) LY345899, a folate analog inhibitor of MTHFD2, and (ii) DS44960156, a preferentially selective MTHFD2 inhibitor [4]. LY345899 and DS44960156 have been confirmed to combine with MTHFD2, which abolishes its catalytic function [3, 4]. The substrate-based MTHFD2 inhibitor LY345899 can disturb nicotinamide adenine dinucleotide phosphate (NADPH), redox homeostasis and accelerate cell death under oxidative stress, which has been confirmed to have therapeutic activity against colorectal cancer [5]. DS44960156 had high selectivity (> 18-fold) for MTHFD2 over methylenetetrahydrofolate dehydrogenase 1 (MTHFD1), with a molecular weight of less than 400, and excellent ligand efficiency. It owns a weak inhibitory effect on MTHFD1 and is less likely to cause safety hazards [4]. All these points indicate that DS44960156 may function as a crucial inhibitor of MTHFD2. Through treatment with two drugs, we may be able to illustrate phenomena by MTHFD2 inhibition and refine Tanaka, Kazuhiro et al.’s research, which promotes development of targeted MTHFD2 therapy and clinical translation of the related mechanism.

**Fig. 1:**
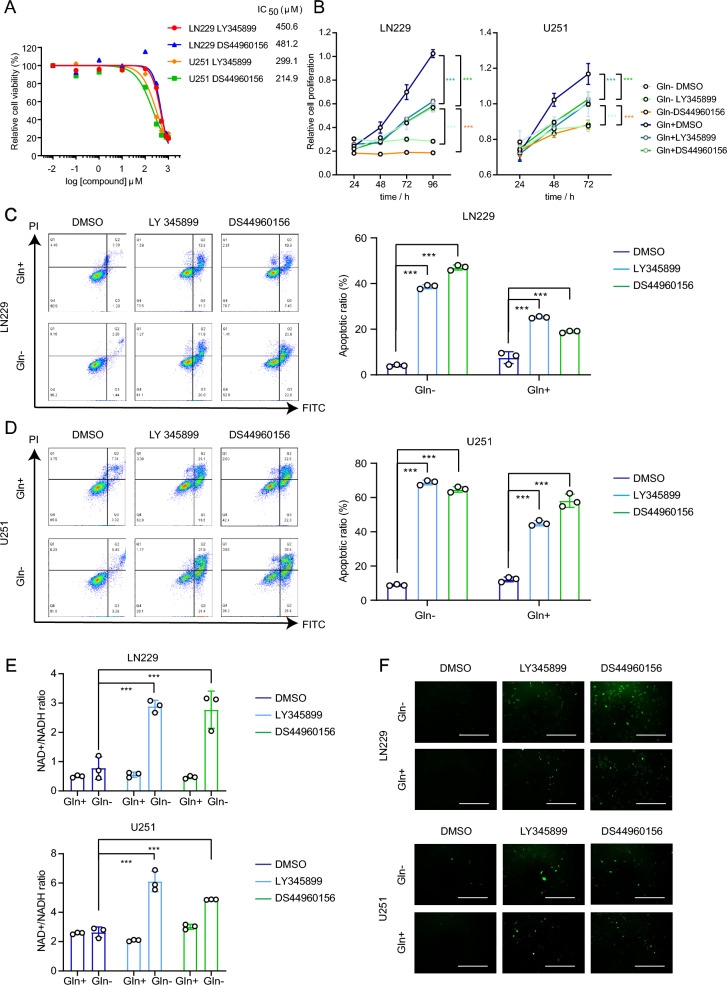
**A** IC50 measurement of LY345899 and DS44960156. **B** LN229 and U251 cells were treated with two types of MTHFD2 inhibitors and DMSO for 24 h, which was changed to medium with or without glutamine on Day 1. The cell number over time represents the mean ± SD of four independent experiments (statistically significant with ***p < 0.001). **C** and **D** Flow cytometric analysis showing the impact of LY345899 and DS44960156 on apoptosis of LN229 and U251 cells with or without glutamine. The right panels show quantification of the apoptosis rate (statistically significant with ***p < 0.001). **E** The NAD + /NADH ratio in LN229 and U251 cells treated with MTHFD2 inhibitors. Data represent the mean ± SD of three independent experiments (statistically significant with ***p < 0.001). **F** Representative fluorescence microscopy images of ROS signals in LN229 and U251 cells. Scale bar: 100 μm

To ensure that druggable interpretation by LY345899 and DS44960156 block MTHFD2 inhibition of glioma cell proliferation, we performed in vitro experiments to show their functions. First, we measured the IC50 of the two drugs (Fig. 1A). Next, we used LY345899 and DS44960156 at suitable doses to treat the glioblastoma cell lines LN229 (LY345899: 500 μM; DS44960156: 500 μM) and U251 (LY345899: 300 μM; DS44960156: 300 μM) with or without glutamine and checked cell viability. Functional blockade of MTHFD2 by intervention with LY345899 and DS44960156 inhibited proliferation of all GBM cells and enhanced the antiproliferative effect under glutamine starvation (Fig. 1B). Moreover, blockade of MTHFD2 function effectively sensitized LN229 and U251 cells to glutamine starvation-mediated cell death (Fig. 1C and D). Serine-derived one-carbon units are used in the folate cycle, which is essential for nucleotide synthesis and production of NADPH, Nicotinamide adenine dinucleotide (NADH), and adenosine triphosphate (ATP). MTHFD1-dependent reduction of 10-formyl-THF to 5,10-methylene-THF is energetically facilitated by a higher NADPH/NADP + ratio in the cell membrane, whereas the MTHFD2 reaction is facilitated by a mitochondrial driven by a higher oxidative oxidation potential, favoring utilization of NAD + by MTHFD2[2]. To further demonstrate the effect of LY345899 and DS44960156 interventions on the function of MTHFD2 in redox homeostasis, we analyzed the intracellular NAD + /NADH ratio. Glutamine starvation resulted in an increased NAD + /NADH ratio in LN229 and U251 cells for redox maintenance. Importantly, pharmacological intervention of MTHFD2 led to an increase in the NAD + /NADH ratio (Fig. 1E). This suggests that the function of MTHFD2 is restricted by inhibiting application of NAD + to maintain redox, leading to extensive cell death upon glutamine starvation. To assess a direct link to increased cytotoxicity via redox status, we performed reactive oxygen species (ROS) assays in GBM cells and found glutamine-deficient ROS signaling to be increased following inhibition of MTHFD2 function (Fig. 1F).

Our data show for the first time that pharmacological intervention via LY345899 and DS44960156 in a low-glutamine tumor microenvironment can effectively inhibit MTHFD2 function and suppress serine-mediated one-carbon metabolism in glioma, which promotes cell death. This further demonstrates that MTHFD2 maintains the cellular energy supply in GBM cells by mediating redox maintenance of one-carbon metabolism; application of LY345899 and DS44960156 will facilitate clinical translation of this theory. The Topological Polar Surface Area (TPSA) scores of LY345899 (TSPA: 211.05) and DS44960156 (TSPA: 87.82) indicates that the two compounds have generally moderate to low blood–brain barrier (BBB) penetration, according to the Molinspiration Cheminformatics (http://www.molinspiration.com) prediction. This challenges the administration of both medications in glioma drug therapy[1]. Because of that, optimization of their chemical structures, as well as potential treatment breakthroughs for MTHFD2, urge for further investigation. Also, the development of drug delivery systems, like as nanoparticles, could allow these medications to be utilized in clinical therapy for GBM. Summarily, targeting against MTHFD2 would be a promising therapy for GBM.

## Data Availability

All data generated or analyzed during this study are included in this article.

## Abbreviations

MTHFD2: Methylenetetrahydrofolate dehydrogenase 2
GBM: Glioblastoma multiforme
NADH: Nicotinamide adenine dinucleotide
NADPH: Nicotinamide adenine dinucleotide phosphate
MTHFD1: Methylenetetrahydrofolate dehydrogenase 1
ATP: Adenosine triphosphate
TPSA: Topological polar surface area
BBB: Blood–brain barrier
